# Race, Ethnicity, Sex, Sexual Orientation, and Discrimination in the Adolescent Brain Cognitive Development Study

**DOI:** 10.1001/jamanetworkopen.2025.10799

**Published:** 2025-05-16

**Authors:** Zhenqiang Zhao, Jinjin Yan, Yijie Wang, Cindy H. Liu, Lijuan Wang, Heining Cham, Tiffany Yip

**Affiliations:** 1 Department of Psychology, Fordham University, Bronx, New York; 2 Department of Human Development and Family Studies, Michigan State University, East Lansing; 3 Department of Pediatric Newborn Medicine and Psychiatry, Brigham and Women’s Hospital, Harvard Medical School, Boston, Massachusetts; 4 Department of Psychology, University of Notre Dame, Notre Dame, Indiana

## Abstract

**Importance:**

Children are disproportionately exposed to sexual orientation–based discrimination and ethnic or racial discrimination due to intersections of sexual orientation, ethnicity, race, and assigned sex at birth. Yet, there is sparse evidence in clinical settings.

**Objective:**

To investigate how social strata of sexual orientation, ethnicity, race, and assigned sex at birth intersect and are associated with experiences of sexual orientation–based discrimination and ethnic or racial discrimination.

**Design, Setting, and Participants:**

This survey study included data retrieved from children enrolled in the Adolescent Brain Cognitive Development Study between 2016 and 2020. Children were recruited from 21 study sites across 17 states. Eligible participants were between ages 9 and 11 years at recruitment. Baseline data (2016-2018), first-year follow-up data (2017-2019), and second-year follow-up data (2018-2020) were included. Analyses were conducted between June and October 2024.

**Main Outcomes and Measures:**

Logistic regressions were conducted to test the association between children’s social strata indicated by intersections of sexual orientation, ethnicity, race, and assigned sex at birth and discrimination based on sexual orientation and ethnic or racial discrimination.

**Results:**

Among 9854 children (mean [SD] age at baseline, 9.5 [0.5] years; 4582 girls [46.5%]; 202 Asian [2.0%], 1488 Black [15.1%], 2030 Latinx [20.6%], 906 multiple races [9.2%], 4921 White [49.9%]), White sexual minority girls reported the highest percentage of sexual orientation–based discrimination (113 of 312 [36.2%]). Ethnically or racially minoritized boys reported the highest percentage of ethnic or racial discrimination (41 of 174 [23.7%]). After accounting for covariates, ethnically or racially minoritized sexual minority girls were less likely to report sexual orientation–based discrimination compared with ethnically or racially minoritized heterosexual boys (odds ratio [OR], 0.60; 95% CI, 0.43-0.85). Ethnically or racially minoritized sexual minority boys (OR, 3.17; 95% CI, 1.71-5.88) and girls (OR, 2.09; 95% CI, 1.47-2.97) were more likely to report ethnic or racial discrimination compared with ethnically or racially minoritized heterosexual boys. Moreover, ethnically or racially minoritized sexual minority boys (OR, 3.39; 95% CI, 1.81-6.34) and girls (OR, 2.24; 95% CI, 1.56-3.21) were more likely to report ethnic or racial discrimination compared with ethnically or racially minoritized heterosexual girls.

**Conclusions:**

In this survey study investigating experience of sexual orientation–based discrimination alongside ethnic or racial discrimination during late childhood, findings highlighted that intersections of sexual orientation, ethnicity, race, and assigned sex at birth contributed to disproportionate exposures to sexual orientation–based discrimination and ethnic or racial discrimination among children. These findings provide valuable insight into intersectional experiences of discrimination among children.

## Key Points

**Question:**

Is the intersection of a child’s sexuality, ethnicity, race, and assigned sex at birth associated with discrimination based on sexual orientation, ethnicity, or race?

**Findings:**

This survey study of 9854 children enrolled in a cognitive development study highlighted that girls from minoritized ethnic or racial groups and with a minority sexuality were less likely to experience sexual orientation–based discrimination compared with boys from these groups. Children from groups minoritized by ethnicity or race and sexuality were more likely to experience ethnic or racial discrimination compared with children from the same groups who were ethnically or racially minoritized and heterosexual.

**Meaning:**

These data suggest that intervention programs addressing intersections of multiple forms of vulnerability are essential for mitigating unique discrimination experiences among ethnically or racially minoritized and sexual minority children.

## Teaser

This survey study uses data from the Adolescent Brain Cognitive Development Study to examine the association of intersectional strata of race, ethnicity, sex, and sexual orientation with discrimination based race, ethnicity, and sexual orientation.

## Introduction

Discrimination experiences based on marginalized social strata among youth is a pressing health concern for young people in the US.^1,2^ Systems of oppressions based on heterosexism, racism, and sexism have been continuously exposing sexual minority youth and ethnically or racially minoritized youth to discrimination.^3,4,5^ A study of the National Survey on LGBTQ Youth including adolescents and young adults aged 13 to 24 years found that approximately 73% of sexual and gender minority youth reported experiencing discrimination based on their sexual orientation or gender identity.^5^ Ethnically or racially minoritized youth are more likely to experience racial discrimination, relative to their White peers.^4^ The exposures to discrimination among ethnically or racially minoritized and sexual minority youth negatively impact psychological and physical health,^6,7^ with exposures in childhood having long-lasting effects into adulthood.^8,9^ Using one of the largest contemporary national datasets of youth in the US,^10^ this study focuses on middle childhood to early adolescence, a critical period during which youth begin to understand social norms and expectations concerning sexuality, ethnicity, race, and gender.^11^

Despite being a social determinant of youth health,^6,12^ significant gaps persist in research focused on understanding disproportionate exposures to discrimination experiences across social strata. For example, minority stress theory suggests that social and structural factors (eg, discrimination, stigma, social marginalization) can contribute to health disparities among individuals from marginalized groups.^13^ Yet, discrimination experiences are not always based on single aspect of marginality, defined as the status of individuals or group being marginalized, in many cases youth are subjected to unfair treatment based on multiple aspects of social marginality.^14^ An intersectionality framework recognizes that experiences of discrimination are situated across multiple social strata, such as ethnicity, race, assigned sex at birth, and sexual orientation.^15,16,17^ That is, a particular form of discrimination associated with a specific social stratification or system of oppression (eg, ethnic and/or racial discrimination) can be shaped and influenced by other intersecting systems of oppression (eg, sexism and heterosexism), which leads to disproportionate exposures of a particular form of discrimination.

Compared with those occupying single forms of marginalized social strata, individuals with multiple marginalized forms of social strata (eg, ethnically or racially minoritized heterosexual individual vs ethnically or racially minoritized and sexual minority individuals) experience poorer health outcomes.^2,18,19,20^ For example, ethnically or racially minoritized sexual minority youth, or sexual minority female youth reported more frequent sexual orientation–based discrimination compared with non-Hispanic White sexual minority youth or sexually minority male youth.^21,22,23^ These studies suggest that intersections of marginalized social strata may increase the exposure to discrimination within our current stratified systems plagued with structural oppressions (eg, racism or sexism). Thus, investigating experiences of discrimination across social strata has critical health implications for supporting youth who have been disproportionately exposed to various forms of discrimination experiences.

Advancing health equity requires a concerted focus on discrimination experiences among youth with multiple marginalized social strata. Thus, the current study applies an intersectionality framework to address a critical gap related to the experience of discrimination among children. This study aimed to: (1) document the experience of sexual orientation–based discrimination and ethnic and/or racial discrimination among children at the intersections of sexual orientation, ethnicity, race, or assigned sex at birth; (2) unveil how exposure of sexual orientation–based discrimination or ethnic and/or racial discrimination differs by such social stratification.

## Methods

This study analyzed data from the first 3 waves of the Adolescent Brain Cognitive Development (ABCD) Study^24^ data release 5.1, including baseline (11 876 participants), 1-year follow-up (11 225 participants), and 2-year follow-up (10 414 participants) survey results. The ABCD study is an ongoing 10-year longitudinal cohort study examining children’s brain development and health outcomes from late childhood to adolescence in the US. The research team recruited children between 9 to 11 years old and their parents or caregivers from 2016 to 2018 across 21 study sites in the US using stratified probability sampling to reflect sociodemographic diversity of the US population.^10^ Written informed consent from the primary caregiver and assent from the children were obtained. The research procedure was approved by the institutional review board at each data collection site. This study follows the American Association for Public Opinion (AAPOR) reporting guideline for survey studies by providing detailed information on the survey methodology, including sampling design, data collection procedures, and measure descriptions. Among 11 876 children who were invited to the ABCD study at baseline, children who opted out of both 1- and 2-year follow-up data collection were excluded from the current study (1462 participants). For families with twins, triplets, and nontwin siblings, only 1 child from each family was selected at random for this study. After excluding 560 siblings, the final analytic sample included 9854 children.

### Measures

#### Intersectional Social Strata

This study included 3 key sociodemographic characteristics—ethnicity and race, sex at birth, and sexual orientation. Ethnicity and race were assessed with 2 questions at baseline. The first question asked participants’ caregivers to respond to the prompt, “What race do you consider the child to be?” and “Do you consider the child Hispanic/Latino/Latina?” Responses had several orders of subgrouping, first categorized as Asian (Asian Indian, Chinese, Filipino, Japanese, Korean, Vietnamese, and other), Black (or African American), Latinx (Hispanic, Latino, or Latina), White, and other (American Indian or Native American, Alaska Native, Guamanian, Native Hawaiian, Samoan, or other Pacific Islander) (eTable 1 in Supplement 1).

Sex at birth was assessed with a single question at baseline. Participants’ caregivers responded to the prompt, “What sex was the child assigned at birth, on the original birth certificate?” Response options included “male,” “female,” “refuse to answer,” and “don’t know.”

Regarding sexual orientation, this study collected participant reports of sexual orientation at the most recent wave (ie, 2-year follow-up). Sexual orientation was assessed with a single question, “Are you gay or bisexual?” Response options included “yes,” “maybe,” “no,” “I do not understand this question,” and “refuse to answer.” This item has been used in the previous studies to measure children’s sexual identity.^25^

Ethnicity and race, sex at birth, and sexual orientation were further recoded to identify different groups of intersectional sociodemographic characteristics. First, participants’ ethnicity and race were dichotomized as non-Hispanic White (ie, White and not Hispanic, Latino, or Latina) and ethnically or racially minoritized group (ie, all remaining participants). Sex at birth was dichotomized as male and female. Sexual orientation was dichotomized as heterosexual (ie, answered “no” to the question) group and sexual minority (ie, answered “yes” or “maybe” to the question).

To investigate disparities in experiencing sexual orientation–based discrimination, we first stratified children into heterosexual and sexual minority children. Sexually minority children were then further stratified based on ethnicity and race and assigned sex at birth (ie, White sexual minority boys, White sexual minority girls, sexual minority boys from racial or ethnic minority groups, and sexual minority girls from racial or ethnic minority groups), whereas heterosexual children were not further stratified given that heterosexual children are less likely to be discriminated due to their sexual orientation in the heteronormative society (eFigure 1 in Supplement 1).

Similarly, to investigate disparities in experiencing ethnic or racial discrimination, we stratified children into White and ethnically or racially minoritized children. Ethnically or racially minoritized children were further stratified based on sexual orientation and assigned sex at birth (ie, ethnically or racially minoritized heterosexual boys, ethnically or racially minoritized heterosexual girls, ethnically or racially minoritized sexual minority boys, and ethnically or racially minoritized sexual minority girls), whereas White children were not further stratified given that White children are less likely to be discriminated due to their race in the White-dominant country (eFigure 2 in Supplement 1).

#### Discrimination

At the first- and second-year follow-up surveys, 2 questions assessed discrimination.^26^ The first question asked about children’s experience of sexual orientation–based discrimination (ie, “In the past 12 months, have you felt discriminated against: because someone thought you were gay, lesbian, or bisexual?”) The second question asked about participants’ experience of ethnic or racial discrimination (ie, “In the past 12 months, have you felt discriminated against: because of your race, ethnicity, or color? Definition of ethnicity: groups of people who have the same customs, or origin.”) In both questions, participants were prompted to answer “yes,” “no,” “don’t know,” or “refused to answer.” Given that discrimination is found to be a low-frequency experience,^27^ each sexual orientation–based discrimination and ethnic or racial discrimination at the first-year follow-up wave were combined with the second-year follow-up wave and dichotomized to indicate whether children experienced sexual orientation-based discrimination or ethnic or racial discrimination, respectively, in 2 years (ie, answered “no” in both waves or answered “yes” in either wave).

#### Covariates

Empirically informed covariates were selected from the caregivers’ surveys at baseline: children’s age, generational status, family structure, and socioeconomic status.^1,28,29,30^ Immigration status was assessed with 3 questions asking about the child’s, biological father’s, and biological mother’s country of birth from a list of countries (eg, “In which country was the child born?”). Then, children’s generational status was recoded as third generation (ie, both biological parents and child were born in the US) or other immigrant statuses (ie, either biological parents or child was born outside of the US). Family structure captures whether children were living with both of their parents (yes or no). Socioeconomic status was created as a latent factor score capturing parental education (λ = 0.71), employment status (λ = 0.48), and family economic hardship (λ = -0.39) such that higher scores of socioeconomic status indicate higher levels of parental education, being employed, and lower levels of family economic hardship.

### Statistical Analysis

All analyses were conducted in R software version 4.1.3 (R Project for Statistical Computing). A series of logistic regressions using generalized linear modeling investigated disparities of discrimination (ie, ethnic, racial, or color discrimination, sexual orientation–based discrimination) by demographic reports of sexual orientation, ethnicity or race, and assigned sex at birth. The first set of analyses examined the association between social strata and experiences of sexual orientation–based discrimination. Comparing heterosexual children and sexual minority children across various social strata (ie, ethnicity or race and assigned sex at birth), we examined the odds of experiencing sexual orientation–based discrimination. Next, we examined sexual orientation–based discrimination among sexual minority children by comparing sexual minority children with fewer minoritized social strata with sexually minoritized children with more minoritized social strata (eg, whether sexual minority girls from racial or ethnic minority groups are more likely to report sexual orientation–based discrimination compared with White sexual minority boys).

The second set of analysis examined the association between social strata and ethnic or racial discrimination. Comparing White children and ethnically or racially minoritized children, we examined ethnic and racial discrimination. Then, we examined the ethnic or racial discrimination among ethnically or racially minoritized children by comparing ethnically or racially minoritized children with fewer minoritized social strata with ethnically or racially minoritized children with more minoritized social strata (eg, whether sexuality minority girls from minoritized ethnic and racial groups are more likely to report ethnic or racial discrimination compared with heterosexual boys).

The significance level was determined with the corrected *P* value using Benjamini and Hochberg postestimation to reduce the ratio of the number of false-positive results to the number of total positive test results.^31^ Missingness also included responses of “refuse to answer” and “don’t know” in all questions. Missingness proportions of key variables ranged from 1.4% (ethnicity or race) to 16.9% (sexual orientation-based discrimination at 2-year follow-up). The correlation between missingness and key variables were small (*r* < .19), indicating that missingness is weakly related to observed data, which supports missing conditionally at random mechanism.^32^ Missing data were handled with multiple imputation in Blimp 3.0. Bayesian estimation was used to estimate missing data (2 chains, 30 000 iterations, 2500 burn, 20 imputations). The default noninformative priors implemented in Blimp 3.0 were used so that priors had little influence on the results.^33^ The potential scale reduction (PSR) values were smaller than 1.05 for all the fitted models, which indicate that model was converged.^33^ Covariates were accounted for in all models.

## Results

Among 9854 children in the analytic sample (mean [SD] age, 9.5 [0.5] years), most of children identified as cisgender male (ie, male children whose gender identity aligns with their assigned sex at birth; 5173 [52.5%]) or cisgender female (ie, female children whose gender identity aligns with their assigned sex at birth; 4582 [46.5%]), and 99 (1.0%) of the sample identified as transgender. The majority of the children were White (4921 [49.9%]), followed by Latinx (2030 [20.6%]), Black (1488 [15.1%]), multiple races (906 [9.2%]), and Asian (202 [2.0%]). In addition, 7139 children (72.4%) were living with both parents; 7426 children and their parents (75.4%) were born in the US. Report of sexual orientation-based discrimination in the past 24 months was highest among White sexual minority girls, followed by ERM SM boys, White SM boys, and ERM SM girls (Table 1). Reports of ethnic or racial discrimination in the past 24 months was highest among sexual minority boys from minoritized racial or ethnic groups (41 of 174 [23.6%]), followed by sexual minority girls from minoritized racial or ethnic groups (26 of 155 [16.8%]), heterosexual girls from minoritized racial or ethnic groups (186 of 1736 [10.7%]), and heterosexual boys from minoritized racial or ethnic groups (212 of 2000 [10.6%]) (Table 2).

**Table 1.** Children Reporting Sexual Orientation–Based Discrimination at First- and Second-Year Follow-Up Surveys by Intersections of Social Stratification

Subgroup	Sample size	Children, No. (%)		
		First-y follow-up	Second-y follow-up	Total
Heterosexual children	7871	236 (3.0)	252 (3.2)	409 (5.2)
White sexual minority boy	68	14 (20.6)	15 (22.1)	21 (30.9)
White sexual minority girl	312	41 (13.1)	111 (35.6)	113 (36.2)
Sexual minority boy from racial or ethnic minority group	174	38 (21.8)	50 (28.7)	62 (35.6)
Sexual minority girl from racial or ethnic minority group	155	14 (9.0)	32 (20.7)	36 (23.3)
Full sample^a^	9854	343 (3.5)	460 (4.7)	641 (6.5)

^a^ Among the final analytic sample (9854 children), 1274 participants were not identifiable for social stratification groups due to missingness in sexual orientation.

**Table 2.** Children Reporting Ethnic or Racial Discrimination at First- or Second-Year Follow-Up Surveys by Intersections of Social Stratification

Subgroup	Sample size	Children, No. (%)		
		First-y follow-up	Second-y follow-up	Total
White children	4515	104 (2.3)	113 (2.5)	185 (4.1)
Heterosexual boy from racial or ethnic minority group	2000	128 (6.4)	136 (6.8)	212 (10.6)
Heterosexual girl from racial or ethnic minority group	1736	113 (6.5)	121 (7.0)	186 (10.7)
Sexual minority boy from racial or ethnic minority group	174	24 (13.8)	28 (16.1)	41 (23.6)
Sexual minority girl from racial or ethnic minority group	155	11 (7.1)	20 (12.9)	26 (16.8)
Full sample^a^	9854	380 (3.9)	418 (4.2)	650 (6.6)

^a^ Among the final analytic sample (9854 children), 1274 participants were not identifiable for social stratification groups due to missingness in sexual orientation.

### Social Strata and Sexual Orientation–Based Discrimination

**Figure 1.**
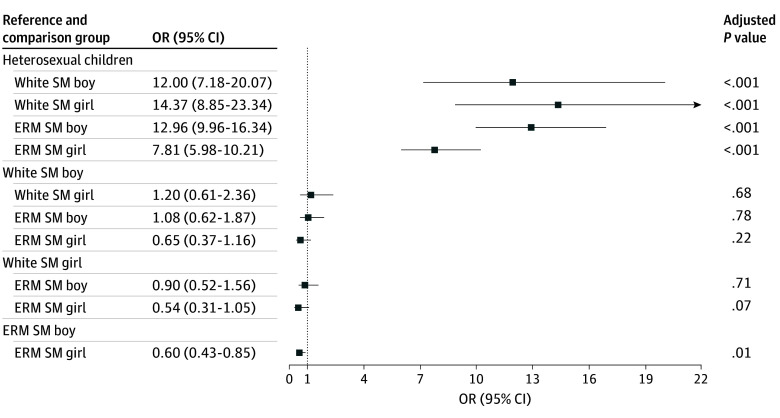
Results for Logistic Regression of Intersectional Sexual Minority Groups on Sexual Orientation–Based Discrimination OR indicates odds ratio. Sample sizes for each social strata group are as follows: 7756 heterosexual children, 68 White sexual minority boys, 312 White sexual minority girls, 174 sexual minority boys from sexual or ethnic minority groups, and 155 sexual minority girls from sexual or ethnic minority groups. Children’s age, generational status, family structure, and socioeconomic status were controlled for logistic regression (eTable 3 in Supplement 1). Heterosexual sample includes non-Hispanic White boys, non-Hispanic White girls, boys from sexual or ethnic minority groups, and girls from sexual or ethnic minority groups (eTable 5 in Supplement 1).

Based on logistic regressions, sexual minority children including White sexual minority boys (odds ratio [OR], 12.00; 95% CI, 7.18-20.07), White sexual minority girls (OR, 14.37; 95% CI, 8.85-23.34), sexual minority boys from minoritized racial or ethnic groups (OR, 12.96; 95% CI, 9.96-16.34), and sexual minority girls from minoritized racial or ethnic groups (OR, 7.81; 95% CI, 5.98-10.21), respectively, were more likely to report sexual orientation-based discrimination compared with heterosexual children (Figure 1). Among sexual minority children, girls from minoritized racial or ethnic groups were less likely to report sexual orientation–based discrimination compared with boys (OR, 0.60; 95% CI, 0.43-0.85). No other significant group differences were found. Among the covariates, children who live with both parents (OR, 0.74; 95% CI, 0.61-0.90) and children from families with higher socioeconomic status (OR, 0.70; 95% CI, 0.63-0.77) were less likely to experience sexual orientation-based discrimination (eTable 3 in Supplement 1).

### Social Strata and Ethnic or Racial Discrimination

**Figure 2.**
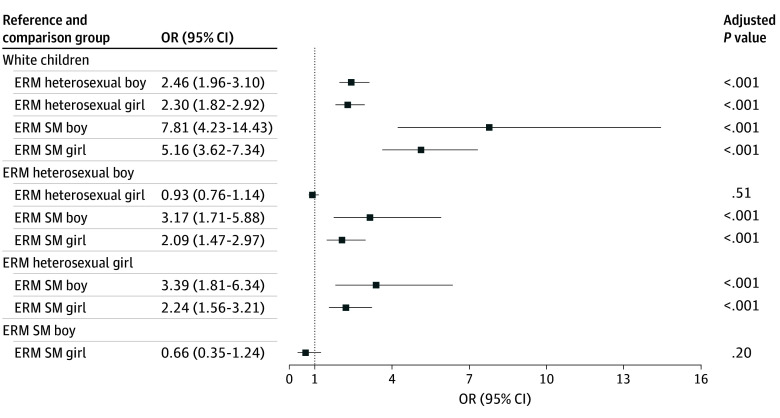
Results for Logistic Regression of Intersectional Ethnic or Racial Minority Groups on Ethnic, Racial, or Color Discrimination OR indicates odds ratio. Sample sizes for each social strata group are as follow: 4980 White children, 2000 heterosexual boys from sexual or ethnic minority groups, 1735 heterosexual girls from sexual or ethnic minority groups, 174 sexual minority boys from sexual or ethnic minority groups, and 155 sexual minority girls from sexual or ethnic minority groups. Children’s age, generational status, family structure, and socioeconomic status were controlled for logistic regression (eTable 4 in Supplement 1). Non-Hispanic White sample includes heterosexual boys, heterosexual girls, sexual minority boys, and sexual minority girls.

Based on logistic regressions, children from minoritized racial or ethnic groups, including heterosexual boys (OR, 2.46; 95% CI, 1.96-3.10), heterosexual girls (OR, 2.30; 95% CI, 1.82-2.92), sexual minority boys (OR, 7.81; 95% CI, 4.23-14.43), and sexual minority girls (OR, 5.16; 95% CI, 3.62-7.34), respectively, reported higher odds of ethnic or racial discrimination compared with White children (Figure 2). Among children from minoritized racial or ethnic groups, sexual minority boys (OR, 3.17; 95% CI, 1.71-5.88) and sexual minority girls (OR, 2.09; 95% CI, 1.47-2.97) reported higher odds of ethnic or racial discrimination compared with heterosexual boys. Moreover, sexual minority boys from minoritized racial or ethnic groups (OR, 3.39; 95% CI, 1.81-6.34) and sexual minority girls from minoritized racial or ethnic groups (OR, 2.24; 95% CI, 1.56-3.21) reported higher odds of ethnic or racial discrimination compared with heterosexual girls from minoritized racial or ethnic groups. Among the covariates, children who live with both parents (OR, 0.76; 95% CI, 0.63-0.92) and parents with higher socioeconomic status (OR, 0.80; 95% CI, 0.73-0.88) reported lower odds of ethnic or racial discrimination. Older children (OR, 1.18; 95% CI, 1.01-1.36) and children whose family members were born in the US (OR, 1.23; 95% CI, 1.02-1.48) reported higher odds of ethnic or racial discrimination (eTable 3 in Supplement 1).

## Discussion

Discrimination is an important social determinant of health among children with marginalized social identities.^22^ Focusing on ethnic or racial discrimination and sexual orientation–based discrimination, this study investigated how children who occupy various nexuses of marginalized social strata experience singular and multiple forms of status-based discrimination.

First, we explored exposure to sexual orientation–based discrimination among sexual minority children across ethnicity, race, and assigned sex at birth. Occupying several marginalized social strata is associated with exposures to interlocking systems of structural oppression (eg, racism, sexism, heterosexism), which contributes to more health risks.^20,34^ Contrary to prior data that suggests exposure to more structural biases and stigmas may increase the chance of encountering hostile and unfair incidents,^21^ the current analyses suggest that sexual minority boys from minoritized racial or ethnic groups reported higher sexual orientation–based discrimination odds compared with sexual minority girls from minoritized racial or ethnic groups. Specifically, whereas both sexual minority boys and girls from minoritized racial or ethnic groups are likely to face interlocking racism and heterosexism, girls are also exposed to sexism, which may exacerbate the experience of sexual orientation-based discrimination among this subgroup. However, these data suggest that sexual minority girls from minoritized racial or ethnic groups reported lower odds of sexual orientation–based discrimination. Of note, the significant sex-based differences in sexual orientation–based discrimination between sexual minority boys from minoritized racial or ethnic groups and sexual minority girls from minoritized racial or ethnic groups were not found between White sexual minority boys and sexual minority girls from minoritized racial or ethnic groups, suggesting that sex differences in sexual orientation–based discrimination may also be tied to ethnicity or race. It is possible that the racial or ethnic minority identity exacerbated the conflicting cultures between sexual minority communities and gender norms. For example, it is possible that masculinity (a dominant gender norm among male children), is more salient among boys from minoritized racial or ethnic groups than femininity is among girls. Thus, ethnicity or race exacerbated the interference of violation of gender norms with homosexuality (eg, not conforming to traditional masculinity such as feminine gender expression among gay men) makes sexual minority boys from minoritized racial or ethnic groups more likely to be targeted for sexual orientation–based discrimination, increasing exposures to prejudice and unfair treatment among sexual minority boys from minoritized racial or ethnic groups compared with girls.^35^ This finding also supported by a previous study of 1518 secondary school students in the Netherlands that found sexual minority boys from minoritized racial or ethnic groups reported lower acceptance of same-sex sexuality and gender nonconformity compared with girls.^36^

We also found that sexual minority children from minoritized racial or ethnic groups had higher odds of ethnic or racial discrimination compared with heterosexual boys and girls. Unlike other minoritized strata (eg, ethnicity, race, gender), the invisible and concealable nature of one’s sexual orientation (ie, the ability to disclose or conceal sexual orientation to others) influences sexual minority children’s social relationship.^37^ Concealing sexual orientation may increase concealment stress such that children become more vigilant about their social context to avoid the disclosure of their sexual orientation.^37^ Thus, it is possible that the ability for evaluating hostility against sexual minority populations in their social context allows children to identify incidents of other forms of unfair treatment such as ethnic or racial discrimination. On the other hand, disclosing sexual orientation may increase the risk of facing systemic heterosexism (eg, victimization), which reinforces their vigilance of unfair treatment.^38^ Thus, it is possible that identifying as sexual minoritization–exposed children in more hostile social contexts and intersecting systems of oppression (ie, racism and heterosexism), increasing exposures to ethnic or racial discrimination compared with White heterosexual children.

This study also supports previous research that found sexual minority children have higher odds of sexual orientation–based discrimination compared with heterosexual children. Similarly, racial or ethnic minority identity children have higher odds of ethnic or racial discrimination compared with White children. Youth who are socially marginalized are exposed to structural biases such as racism or heterosexism in various contexts such as education and health care.^3,39^ These structures collectively increase exposure to discrimination when compared with heterosexual or White children. Using a diverse, national probability-based sample of children from ages 11 to 14 years old, this study noted the emergence of social status–based discrimination in late childhood highlighting the importance of preventative efforts focused on this age range.

### Limitations

This study has several limitations. First, although gender minority youth comprise 1.4% of the youth population between ages 13 to 17 years old in the US,^40^ we were unable to stratify by gender identity (eg, transmale, transfemale, gender nonbinary) due to the small sample of transgender and gender non-binary children in the sample (ie, 0.9% of total sample). We were also unable to investigate the intersections of ethnicity, race, sex, and sexual orientation with additional social stratifications such as low socioeconomic status family (equivalent with a 4-way interaction) for the sake of interpretability of results. Adding a fourth dimension of intersectionality considerably increases the number of tests conducted for pairwise comparison, thus, making the anticategorical approach to multiple dimensions of intersectionality is more advisable.^41^ Second, we acknowledge that the item used to assess sexual identity can be confusing to children. Although it is possible that children may not understand the meaning of gay or bisexual, our data showed that children’s report of sexual identity was highly correlated with romantic attraction to same-sex peers (*r* = 0.77 for girls and *r* = 0.71 for boys), which supports the validity of the item in this sample. Related, this study recoded responses of “refuse to answer” or “don’t know” to the sexual identity question as missing because there were no follow-up questions available for researchers to further differentiate children who actually did not understand the question or who did not want to answer it to avoid unintended disclosure of their sexual identity. Future studies should utilize additional questions to measure qualitative nuance in children’s sexual identity.^42^ Similarly, there may be reporting bias in sexual orientation–based discrimination. However, this can be further validated as new waves of data from the ABCD study become available, tracking children’s sexual orientation and related discrimination experiences into late adolescence. Third, the subgroup for White sexual minority boys had relatively small sample sizes (ie, 68 participants), which may limit statistical power. Moreover, we were not able to account for heterogeneity within racial or ethnic minority identity or sexual minority children or children who refused to report their sexual orientation due to lack of power for more comprehensive comparisons. Future studies can address this gap by oversampling children who are more likely to be underrepresented in the national probability data (eg, Asian gay children). Last, due to the nature of secondary analysis and infrequent experience of discrimination among younger population, we were not able to apply a developmental framework investigating how social stratification influence discrimination longitudinally. With additional waves of the ABCD study, it will be important to track discrimination experiences from childhood to late adolescence.

## Conclusions

This study makes important contributions to how intersecting social strata are related to minoritized discrimination experiences. That is, while ethnic and racial discrimination differs by stratification based on sexual orientation (eg, sexual minority children from racial or ethnic minority groups are more likely to experience ethnic or racial discrimination compared with heterosexual children), sexual orientation–based discrimination differs by an intersecting stratification of ethnicity, race, and assigned sex at birth (eg, sex difference on sexual orientation–based discrimination was only found among racial or ethnic minority groups but not among White). This study offers novel implications for implementing antidiscrimination policy and prevention and intervention efforts on the systemic level targeted to children from certain intersections of social stratification (eg, discrimination experienced by sexual minority boys based on race, ethnicity, or sexual orientation minority groups) and promoting supportive social contexts and interactions for children with multiple minoritized social statuses.

## Notes

Supplement 1. **eTable 1.** List of Ethnicity or Race Available for Participant Selection and Recoded Groups Used for the Analytic Sample **eTable 2.** Correlations Between Missingness and Key Variables **eTable 3.** Odds Ratio of the Association Between Social Strata and Sexual Orientation-Based Discrimination **eTable 4.** Odds Ratio of the Association Between Social Strata and Ethnic or Racial Discrimination **eTable 5.** Variations in the Experience of Sexual Orientation Discrimination Within Heterosexual Children **eTable 6.** Variations in the Experience of Ethnic, Racial, or Color Discrimination Within White Children **eFigure 1.** Social Strata Based on Sexual Orientation **eFigure 2.** Social Strata Based on Ethnicity and Race

Supplement 2.
Data Sharing Statement
