# Supplementary material for: Race, Ethnicity, Sex, Sexual Orientation, and Discrimination in the Adolescent Brain Cognitive Development Study
Source: JAMA Netw Open. 2025 May 16;8(5):e2510799. doi: 10.1001/jamanetworkopen.2025.10799 (PMC12084841; doi:10.1001/jamanetworkopen.2025.10799)
Supplement: Supplement 1. — eTable 1. List of Ethnicity or Race Available for Participant Selection and Recoded Groups Used for the Analytic Sample eTable 2. Correlations Between Missingness and Key Variables eTable 3. Odds Ratio of the Association Between Social Strata and Sexual Orientation-Based Discrimination eTable 4. Odds Ratio of the Association Between Social Strata and Ethnic or Racial Discrimination eTable 5. Variations in the Experience of Sexual Orientation Discrimination Within Heterosexual Children eTable 6. Variations in the Experience of Ethnic, Racial, or Color Discrimination Within White Children eFigure 1. Social Strata Based on Sexual Orientation eFigure 2. Social Strata Based on Ethnicity and Race [file jamanetwopen-e2510799-s001.pdf]

Supplemental Online Content

Zhao Z, Yan J, Wang Y, et al. Race, ethnicity, sex, sexual orientation, and discrimination in the Adolescent Brain Cognitive Development Study. *JAMA Netw Open.* 2025;8(5):e2510799. doi:10.1001/jamanetworkopen.2025.10799

eTable 1. List of Ethnicity or Race Available for Participant Selection and Recoded Groups Used for the Analytic Sample

eTable 2. Correlations Between Missingness and Key Variables

eTable 3. Odds Ratio of the Association Between Social Strata and Sexual Orientation-Based Discrimination

eTable 4. Odds Ratio of the Association Between Social Strata and Ethnic or Racial Discrimination

eTable 5. Variations in the Experience of Sexual Orientation Discrimination Within Heterosexual Children

eTable 6. Variations in the Experience of Ethnic, Racial, or Color Discrimination Within White Children

eFigure 1. Social Strata Based on Sexual Orientation

eFigure 2. Social Strata Based on Ethnicity and Race

This supplemental material has been provided by the authors to give readers additional information about their work.

© 2025 Zhao Z et al. *JAMA Network Open*.

eTable 1

List of ethnicity/race available for participant selection and recoded groups used for the analytic sample

<table>
<tr><td>Original ethnicity/race</td><td>Lower-order subgroup</td><td>Higher-order subgroup</td></tr>
<tr><td>White</td><td>White</td><td>White</td></tr>
<tr><td>Black/African American</td><td>Black</td><td rowspan="16">Ethnic/racial minority</td></tr>
<tr><td>American Indian, Native American</td><td rowspan="6">Other ethnicity/race</td></tr>
<tr><td>Alaska Native</td></tr>
<tr><td>Native Hawaiian</td></tr>
<tr><td>Guamanian</td></tr>
<tr><td>Samoan</td></tr>
<tr><td>Other Pacific Islander</td></tr>
<tr><td>Asian Indian</td><td rowspan="7">Asian</td></tr>
<tr><td>Chinese</td></tr>
<tr><td>Filipino</td></tr>
<tr><td>Japanese</td></tr>
<tr><td>Korean</td></tr>
<tr><td>Vietnamese</td></tr>
<tr><td>Other Asian</td></tr>
<tr><td>Other race</td><td>Other ethnicity/race</td></tr>
<tr><td>Hispanic/Latino/Latina</td><td>Latinx</td></tr>
</table>

Note. All options except for “Hispanic/Latino/Latina” were provided for the question for parents asking, “*What race do you consider the child to be?*”. Another question asked “*Do you consider the child Hispanic/Latino/Latina?*”

© 2025 Zhao Z et al. *JAMA Network Open*.

eTable 2

Correlations between missingness and key variables

Missingness<br>Variable	Sexual orientation	Race/ ethnicity	Sexual orientation-based discrimination	Racial/ethnic discrimination
Sexual orientation	N.A.	<.001	.03	<.001
Race/ethnicity	-.11	N.A.	-.14	-.15
Assigned sex at birth	.03	-.01	.00	-.01
Sexual orientation-based discrimination	.14	-.01	N.A.	.13
Racial/ethnic discrimination	.19	<.001	.13	N.A.
Age	-.02	-.02	-.05	-.04
Living with Parents	-.08	-.01	-.08	-.09
Socioeconomic status	-.10	.14	-.11	-.14
Generational status	<.001	-.02	-.01	-.03
Missingness	.14	.01	.21	.23

Note. There was no missingness in the assigned sex at birth.

© 2025 Zhao Z et al. *JAMA Network Open*.

eTable 3

Odds ratio of the association between social strata and sexual orientation-based discrimination

	OR	*p*	Adj. *p*	95% *CI [LL,UL]*
Reference: Heterosexual				
Sexual minority, White boy	12.00	<0.001	<0.001	[7.18, 20.07]
Sexual minority ethnic/racial minority boy	14.37	<0.001	<0.001	[8.85, 23.34]
Sexual minority, White girl	12.96	<0.001	<0.001	[9.95, 16.89]
Sexual minority ethnic/racial minority girl	7.81	<0.001	<0.001	[5.98, 10.21]
Covariates				
Age	1.11	0.23	-	[.94, 1.30]
Living with Parents	0.74	0.003	-	[.61, .90]
Socioeconomic status	0.70	<0.001	-	[.63, .77]
Generational status	1.17	0.14	-	[.95, 1.43]

Note. Heterosexual sample includes White boy, White girl ethnic/racial minority boy, and ethnic/racial minority girl (see ETable 5). *OR* represents odds ratio indicating the ratio of the predicted probability of experiencing sexual orientation-based discrimination of the target group compared to the predicted probability of experiencing sexual orientation-based discrimination of the reference group; CI represents confidence intervals. LL and UL represent lower level (2.5%) and upper level (97.5%) of 95% confidence interval. Significance level was determined based on the adjusted p-value (Benjamini & Hochberg, 1995).

© 2025 Zhao Z et al. *JAMA Network Open*.

eTable 4

Odds ratio of the association between social strata and ethnic/racial discrimination

	OR	*p*	Adj. *p*	95% *CI [LL,UL]*
Reference: White				
Heterosexual ethnic/racial minority girl	2.46	<0.001	<0.001	[1.96, 3.10]
Sexual minority ethnic/racial minority boy	2.30	<0.001	<0.001	[1.82, 2.92]
Sexual minority ethnic/racial minority girl	7.81	<0.001	<0.001	[4.23, 14.43]
Sexual minority ethnic/racial minority girl	5.16	<0.001	<0.001	[3.62, 7.34]
Covariates				
Age	1.18	0.03	-	[1.01, 1.36]
Living with Parents	.76	0.005	-	[.63, .92]
Socioeconomic status	.80	<0.001	-	[.73, .88]
Generational status	1.23	0.03	-	[1.02, 1.48]

Note. White sample includes heterosexual boy, heterosexual girl, sexual minority boy, and sexual minority girl (see ETable 6). *OR* represents odds ratio indicating the probability of assigning to the target group compared to probability of assigning to the reference; CI represents confidence intervals. LL and UL represent lower level (2.5%) and upper level (97.5%) of 95% confidence interval. Significance level was determined based on the adjusted p-value (Benjamini & Hochberg, 1995).

* $p < .05$; ** $p < .01$ *** $p < .001$

Significance level was determined based on the adjusted p-value (Benjamini & Hochberg, 1995). Duplicated comparisons are omitted from the table.

* $p < .05$; ** $p < .01$ *** $p < .001$

© 2025 Zhao Z et al. *JAMA Network Open*.

eTable 5

Variations in the experience of sexual orientation discrimination within heterosexual children

	OR	*p*	Adj. *p*	95% *CI [LL,UL]*
Reference: Heterosexual White boy				
Heterosexual White girl	0.90	0.53	.53	[.64, 1.26]
Heterosexual ethnic/racial minority boy	2.79	<0.001	<0.001	[2.12, 3.68]
Heterosexual ethnic/racial minority girl	2.61	<0.001	<0.001	[1.97, 3.46]
Reference: Heterosexual White girl				
Heterosexual ethnic/racial minority boy	3.10	<0.001	<0.001	[2.24, 4.30]
Heterosexual ethnic/racial minority girl	2.90	<0.001	<0.001	[2.09, 4.03]
Reference: Heterosexual ethnic/racial minority boy				
Heterosexual ethnic/racial minority girl	.94	0.51	0.61	[.76, 1.14]
Covariates				
Age	1.18	0.05	-	[1.00, 1.39]
Living with Parents	.75	0.007	-	[.61, .92]
Socioeconomic status	.83	<0.001	-	[.75, .92]
Generational status	1.18	0.09	-	[.97, 1.44]

Note. Heterosexual White boy n = 2335; Heterosexual White girl n = 1793; Heterosexual ethnic/racial minority boy n = 2049; Heterosexual ethnic/racial minority girl n = 1687. *OR* represents odds ratio indicating the probability of assigning to the target group compared to probability of assigning to the reference; CI represents confidence intervals. LL and UL represent lower level (2.5%) and upper level (97.5%) of 95% confidence interval.

© 2025 Zhao Z et al. *JAMA Network Open*.

eTable 6

Variations in the experience of ethnic/racial/color discrimination within White children

	OR	*p*	Adj. *p*	95% *CI [LL,UL]*
Reference: Heterosexual White boy				
Heterosexual White girl	0.91	0.58	0.66	[.43, .82]
Sexual minority White boy	3.43	0.003	<0.001	[1.56, 7.58]
Sexual minority White girl	2.47	<0.001	<0.001	[1.59, 3.84]
Reference: Heterosexual White girl				
Sexual minority White boy	3.78	0.002	<0.001	[1.66, 8.62]
Sexual minority White girl	2.71	<0.001	<0.001	[1.67, 4.40]
Reference: Sexual minority White boy				
Sexual minority White girl	.72	0.43	0.52	[.63, 1.11]
Covariates				
Age	1.12	0.45	-	[.84, 1.25]
Living with Parents	.59	0.005	-	[.58, .96]
Socioeconomic status	.65	<0.001	-	[.68, .87]
Generational status	.99	0.98	-	[.94, 1.57]

Note. Heterosexual, White, boy *n* = 2335; Heterosexual, White girl, *n* = 1793; Sexual minority, White, boy, *n* = 68; Sexual minority White girl, *n* = 312; *OR* represents odds ratio indicating the probability of assigning to the target group compared to probability of assigning to the reference; CI represents confidence intervals. LL and UL represent lower level (2.5%) and upper level (97.5%) of 95% confidence interval. Significance level was determined based on the adjusted p-value (Benjamini & Hochberg, 1995).

© 2025 Zhao Z et al. *JAMA Network Open*.

eFigure 1

Social strata based on sexual orientation

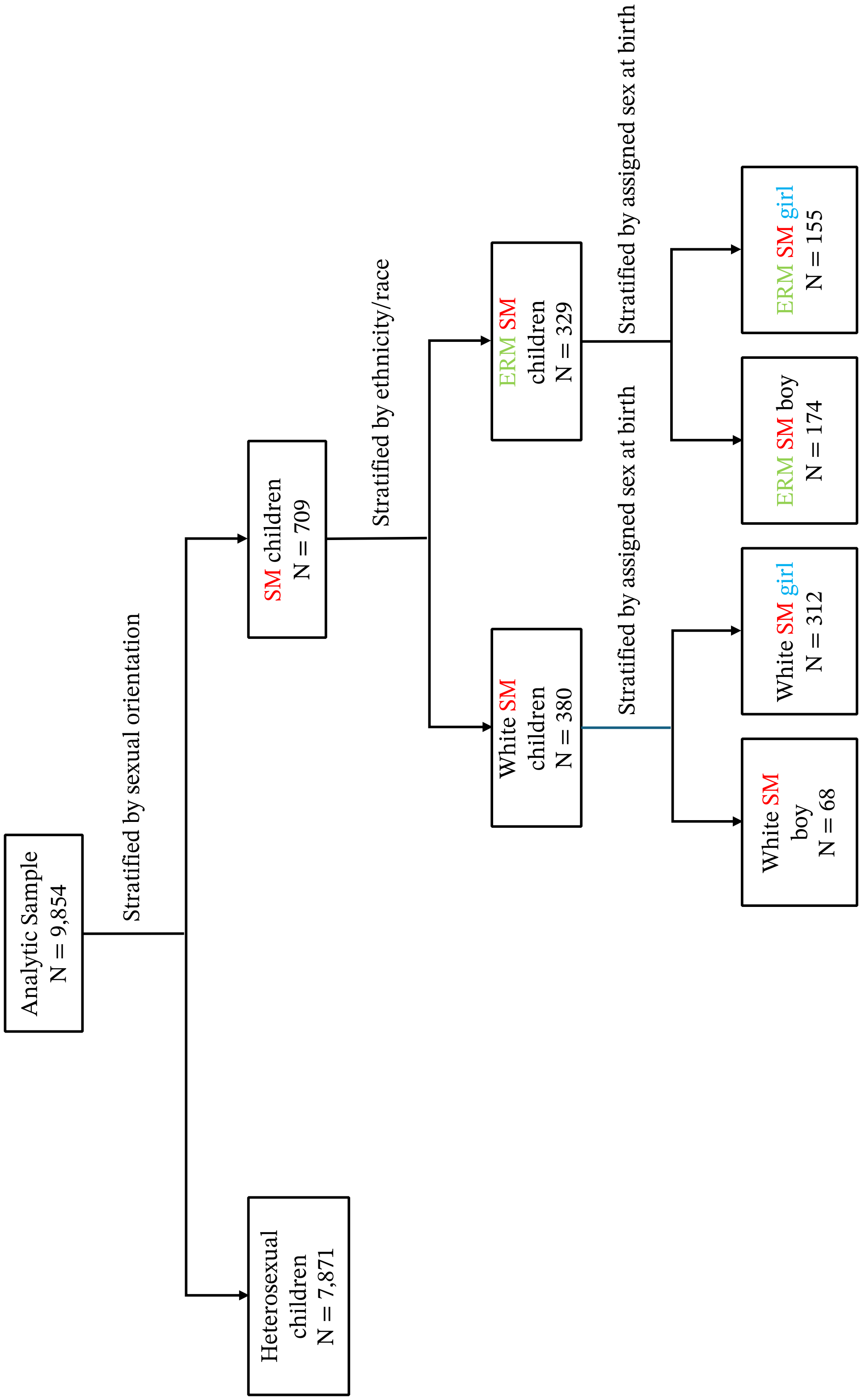

© 2025 Zhao Z et al. *JAMA Network Open*.

eFigure 2

Social strata based on ethnicity/race

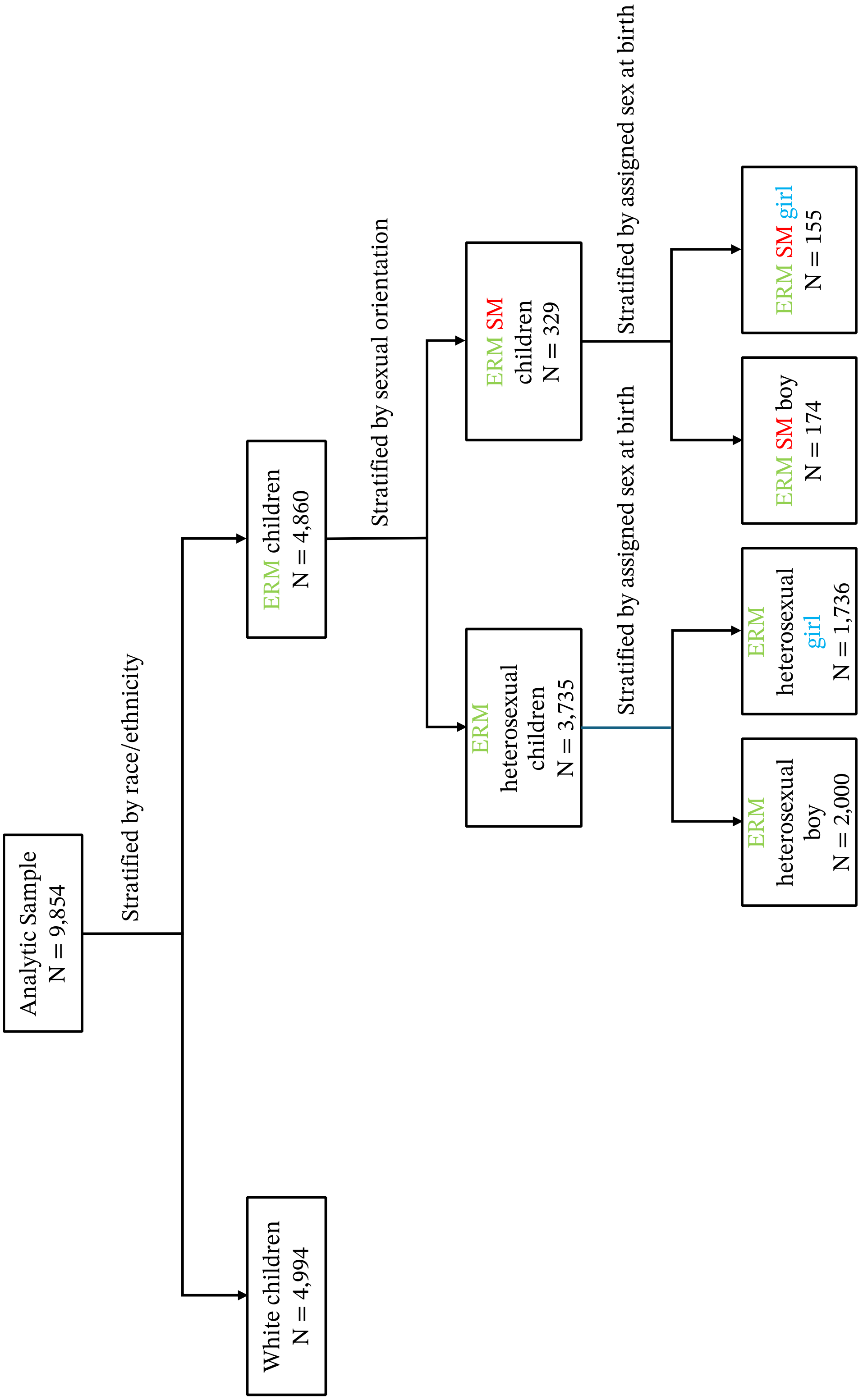

© 2025 Zhao Z et al. *JAMA Network Open*.
