# Supplementary material for: Race, Ethnicity, Sex, Sexual Orientation, and Discrimination in the Adolescent Brain Cognitive Development Study
Source: JAMA Netw Open. 2025 May 16;8(5):e2510799. doi: 10.1001/jamanetworkopen.2025.10799 (PMC12084841; doi:10.1001/jamanetworkopen.2025.10799)
Supplement: Supplement 2. — Data Sharing Statement [file jamanetwopen-e2510799-s002.pdf]

Data Sharing Statement

Zhao. Race, Ethnicity, Sex, Sexual Orientation, and Discrimination in the Adolescent Brain Cognitive Development Study. *JAMA Netw Open*. Published May 16, 2025. doi:10.1001/jamanetworkopen.2025.10799

Data
Data available: No
